# Physical Exercise and Health-Related Quality of Life in Office Workers: A Systematic Review and Meta-Analysis

**DOI:** 10.3390/ijerph18073791

**Published:** 2021-04-05

**Authors:** Thi Mai Nguyen, Van Huy Nguyen, Jin Hee Kim

**Affiliations:** 1 Department of Integrative Bioscience & Biotechnology, Sejong University, 209 Neungdong-ro, Gwangjin-gu, Seoul 05006, Korea; mainguyen@sju.ac.kr; 2 Health Innovation and Transformation Centre, Federation University, Mount Helen, Ballarat, VIC 3350, Australia; h.vannguyen@federation.edu.au; 3 Graduate School of Public Health, St. Luke’s International University, 3-6-2 Tsukiji, Chuo-ku, Tokyo 104-0045, Japan

**Keywords:** physical exercise, health-related quality of life, office worker, meta-analysis

## Abstract

Office workers are at high risk for many chronic diseases, lowering their health-related quality of life (HRQOL). This systematic review and meta-analysis aimed to summarize the effects of physical exercise on HRQOL in office workers with and without health problems using data obtained from randomized controlled trials (RCTs), quasi-experimental, and observational studies. We searched PubMed, Web of Science, Scopus, Cochrane Library, and several grey literature databases, and identified 26 relevant studies for the synthesis. Overall, physical exercise significantly improved general (standardized mean difference (SMD) = 1.05; 95% confidence interval (CI): 0.66 to 1.44) and mental (SMD = 0.42; 95% CI: 0.19 to 0.66) HRQOL in office workers. Compared with healthy office workers, unhealthy office workers experienced greater improvements in general (unhealthy, SMD = 2.76; 95% CI: 1.63 to 3.89; healthy, SMD = 0.23; 95% CI: −0.09 to 0.56) and physical (unhealthy, SMD = 0.38; 95% CI: 0.17 to 0.58; healthy, SMD = −0.20; 95% CI: −0.51 to 0.11) HRQOL. Unsupervised physical exercise significantly improved general and mental HRQOL, while directly supervised physical exercise significantly improved only general HRQOL. Although physical exercise, especially unsupervised physical exercise, should be encouraged to improve HRQOL in office workers, detailed recommendations could not be made because of the diverse exercise types with different intensities. Therefore, further studies are needed to determine the optimal exercise for office workers with different health conditions.

## 1. Introduction

A healthy workplace is defined as a work environment where workers and managers collaborate to improve the health, safety, and well-being of the workforce and thus sustain the productivity of the business [1]. Employers have begun seeking interventions to create healthy workplaces because of the economic benefits and for ethical and legal reasons [1]. Health-related quality of life (HRQOL), a subjective evaluation of personal health status [2], is considered a valuable indicator for healthy workplace assessments. Existing evidence has supported that HRQOL scores are associated with employee productivity, disability, and sickness-related absenteeism [1]. For these reasons, various interventions, such as the provision of health and safety, psychosocial/organizational culture, and personal health-related resources in the workplace, have been developed to improve employees’ HRQOL [1].

Office workers, whose primary tasks generally involve using computers, participating in meetings, giving presentations, reading, and speaking on the phone [3], are at high risk for many chronic diseases, lowering their HRQOL [4]. Such diseases include musculoskeletal disorders, dry eye syndrome, cardio-metabolic diseases, coronary artery disease, metabolic syndrome, and some types of cancer [5,6,7,8]. The development of these diseases may be related to the fact that employees work in a seated position for about two-thirds to three-quarters of their working time, with prolonged and unbroken stretches of 20 min or more [9,10]. Indeed, Hu et al. [11] found that two-hour increments of sitting increased risks of diabetes and obesity by 7% and 5%, respectively. It is widely accepted that workplace health promotion programs should focus on reducing sitting time and increasing workplace-based physical exercise [12].

Physical exercise, known as a cost-effective intervention, is strongly recommended for managing and even preventing many work-related chronic diseases. Recent reviews investigating the effects of physical exercise on the health of office workers [13,14,15] reported significant and protective effects of physical exercise on musculoskeletal pain symptoms (i.e., neck pain and low back pain) [14,15]. While some studies indicated a significant association between physical exercise and HRQOL [14], other studies did not [15]. In addition, previous reviews mostly focused on office workers with musculoskeletal disorders, the most common work-related chronic conditions, which are reported by approximately 50% of office workers in Turkey [16], Ethiopia [17], and Iran [18]. However, little is known about the relation between physical exercise and HRQOL in healthy office workers engaging in sedentary behavior during most of their working time, whose risk of many chronic diseases remains high, or HRQOL in office workers with other types of work-related diseases. To fill in this gap, we conducted this systematic review and meta-analysis using data obtained from randomized controlled trials (RCTs), quasi-experimental, and observational studies to summarize the effects of physical exercise on HRQOL in office workers with and without health problems.

## 2. Materials and Methods

### 2.1. Study Protocol

To summarize the effects of physical exercise on HRQOL in office workers, we followed the Preferred Reporting Items for Systematic Reviews and Meta-Analyses (PRISMA) guidelines in all steps [19], and all procedures were registered at the International Prospective Register of Systematic Reviews (registration number: CRD42020209238).

First, on 27 July 2020, we conducted a literature search without any limitations on publication date or language using different combinations of keywords relating to physical exercise, HRQOL, and office workers. PubMed, Web of Science, Scopus, Cochrane Library, and six grey literature databases including Google, Open Grey, Grey Literature Report, Open Access Theses and Dissertations, Center for Research Libraries, and Dissertations.se were searched. Details about the search terms are presented in Appendix A, Table A1. We also conducted a manual search for citations from the included articles and key review papers to identify additional relevant studies [20].

Second, searched studies were selected based on several criteria. We selected if the study (1) targeted office workers who had spent most of their working time engaging in sedentary behavior; (2) provided data relating to the association between physical exercise and HRQOL; (3) was accessible in full-text format; and (4) was an RCT, quasi-experimental, or observational study. In this review, physical exercise was defined as planned, structured, and repetitive bodily movements that primarily aim to maintain or improve physical health. Exclusion criteria were as follows: (1) studies in which physical exercise was combined with other interventions (i.e., diet modifications or use of height-adjustable desks); (2) studies that recruited both white- and blue-collar workers but did not separate the white-collar worker data; (3) non-original studies, such as reviews, commentaries, or editorials; or (4) ongoing studies that had been registered. If multiple publications contained overlapping data resulting from the same study (i.e., publications reporting subgroups, additional outcomes or exposures outside the scope of an evaluation, or longer follow-up periods), we selected only publications with the largest number of participants or the most recent publication date. The whole process was conducted independently by two authors (T.M.N. and V.H.N.), and screening conflicts were solved by discussion until a consensus was reached. After non-English publications were translated, data for qualitative analysis and meta-analyses were extracted.

### 2.2. Data Extraction for Qualitative Analysis and Meta-Analyses

Data used for qualitative analysis included the name of the first author, year of publication, study design, country, study population, HRQOL questionnaires, and main findings of the included studies. For RCT and quasi-experimental studies, descriptions of interventions were also extracted.

We obtained all quantitative data indicating an association between physical exercise and HRQOL in office workers for meta-analyses. Examples of these data include mean and standard deviation (SD), median and interquartile range of HRQOL score, odds ratio, and effect size Cohen’s d. For studies with missing information, we contacted the corresponding authors via email and requested to receive the full dataset.

Based on relative similarities among the HRQOL questionnaires used in the included studies, we classified the quantitative HRQOL data into general, physical, and mental domains (Appendix A, Table A2). Since a higher score denoted better HRQOL on all HRQOL questionnaires except the Dry Eye-Related Quality of Life Score [21], for purposes of consistency, we multiplied the means of the scores by −1 without changing the SDs [22].

As suggested by the developers of the 36-Item Short-Form Health Survey, we retained two distinct domains of HRQOL, physical and mental, instead of generating a total score when pooling data for the meta-analysis. The physical domain comprises four scales assessing physical function, bodily pain, general health, and role limitations due to physical problems, while the mental domain summarizes four scales including energy/fatigue, emotional well-being, social functioning, and role limitations caused by emotional problems [23].

### 2.3. Risk-of-Bias (ROB) Assessment

Two authors (T.M.N. and V.H.N.) independently assessed the ROB of RCTs, quasi-experimental, and observational studies using the revised Cochrane ROB tool for RCTs (ROB 2.0) [24], ROB in non-randomized studies of interventions (ROBINS-I) [25], and Newcastle-Ottawa quality assessment scale (NOS) [26], respectively. Disagreements were discussed until a consensus was reached. The three ROB assessment tools that were used composed of several categories as follows: ROB 2.0 consists of five potential bias categories that are assessed as low ROB, some concerns, or high ROB using a series of signaling questions. The categories are the randomization process, deviations from intended interventions, missing outcome data, measurement of the outcome, and selection of the reported results [24]. ROBINS-I comprises seven bias categories, namely, baseline confounding, selection of participants, classification of interventions, deviation from intended interventions, missing data, measurement of outcomes, and selection of reported results; each is evaluated as low, moderate, serious, or critical ROB or no information [25]. NOS contains three methodological bias categories, namely, study group selection, comparability among groups, and outcomes of interest. The adapted NOS for cross-sectional studies developed by Herzog et al. [27] and the NOS for cohort studies [26] were applied to evaluate the ROB of cross-sectional and cohort studies, respectively.

Assessment of each above-mentioned category provided the basis for an overall ROB judgment for the included studies.

### 2.4. Meta-Analyses

To indicate the effects of physical exercise on HRQOL in office workers, we computed standardized mean difference (SMD), which is the effect size known in social science as Hedges’s (adjusted) g [22], with a 95% confidence interval (CI). In most cases, Hedges’s g was calculated from the means and SDs of the HRQOL scores. For studies that presented HRQOL scores using box plots, we extracted medians and interquartile ranges of HRQOL scores using Web Plot Digitizer [28]. For studies using medians and interquartile ranges to describe the HRQOL scores, means and SDs were estimated using the method of Wan et al. for both normal and skewed data [29]. For studies using other effect size indicators, we converted them to Hedges’s g using the formula of Borenstein et al. [30] (Appendix A, Table A3). The magnitude of the effect size was defined as small (0.2 to under 0.5), medium (0.5 to 0.8), or large (above 0.8) [31]. The magnitude of heterogeneity was interpreted as follows: low (I^2^ = 0 to 24%), moderate (I^2^ = 25 to 49%), large (I^2^ = 50 to 74%), or extreme (I^2^ = 75 to 100%) heterogeneity [22]. For a multi-arm study, after selecting relevant groups, we treated each intervention–control pair as an individual comparison in a meta-analysis [30].

We conducted meta-analyses for three HRQOL domains (general, physical, and mental) classified by study design. Then, we pooled data obtained only from RCTs to compare the effect of physical exercise on each domain between workers without and with health problems such as musculoskeletal pain, metabolic syndrome, or dry eye syndrome (defined as healthy and unhealthy workers, respectively) and among types of intervention. Due to a great variety of physical exercise interventions used, such as stretching, strengthening, flexibility exercises, etc., we classified them into three groups—directly supervised (i.e., directly guided by professional instructors, certified practitioners, or peer supervisors), indirectly supervised (i.e., periodically sending phone notifications, application reminders, or text messages to participants in the intervention groups), and unsupervised physical exercise (i.e., after being introduced about the physical exercise interventions, participants performed themselves without receiving any periodic supervision or reminders).

To determine the robustness of the outcomes and to confirm our conclusions, we conducted leave-one-out sensitivity analyses, which omitted each study in turn, and the influence analyses proposed by Viechtbauer and Cheung [32], which removed studies that exerted a high degree of influence on the overall effect size. Publication bias was assessed using funnel plots and Egger’s regression asymmetry test, which was used only when at least 10 studies were included in a meta-analysis [22]. We considered *p* < 0.05 in asymmetrical funnel plots to indicate potential publication bias. All statistical analyses were performed using the “meta” and “metafor” packages in R version 3.6.2.

## 3. Results

### 3.1. Selected Studies

**Figure 1:**
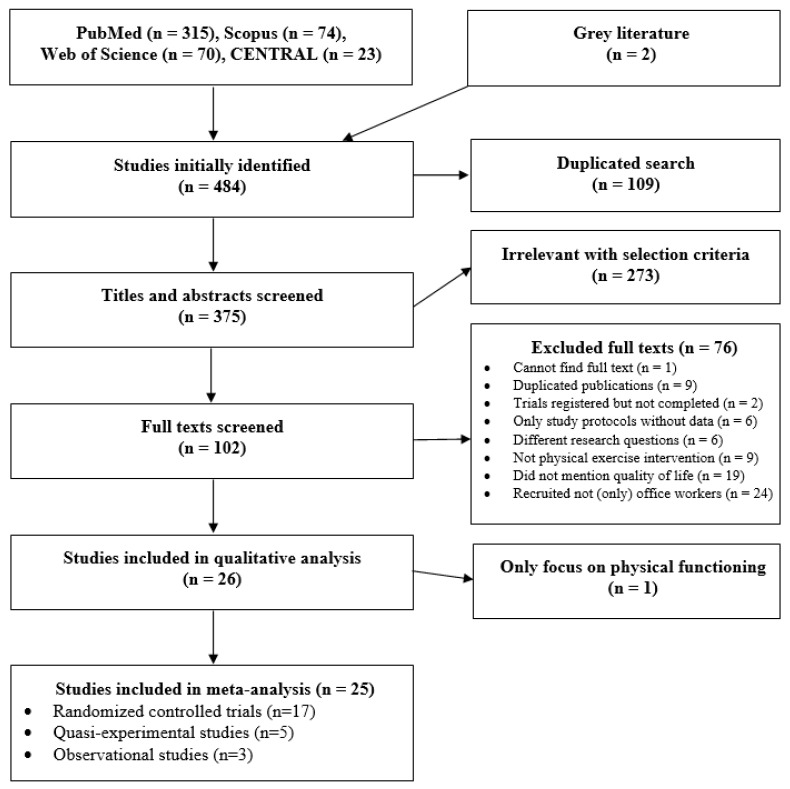
Flow chart of the study selection process.

Figure 1 summarizes the selection process for the 26 studies in this review. After initially identifying 482 records (315, 74, 70, and 23 articles from the PubMed, Scopus, Web of Science, and Cochrane Library, respectively) and two additional grey literature publications, we removed 109 duplicates and then reviewed titles and abstracts of 375 remaining studies. In this step, 273 studies were excluded due to failure to meet our selection criteria. The full texts of 102 potential studies were then extracted and screened for further details. We excluded 76 publications for the following reasons: (1) one unavailable full text; (2) nine duplicated publications; (3) two ongoing trials; (4) six study protocols without mentioning any data; (5) six records focused on different research questions, despite mentioning physical exercise and HRQOL in office workers; (6) 9 and 19 records did not provide data relating to physical exercise and HRQOL, respectively; and (7) 24 studies targeted other subjects, not only office workers. Of 26 studies finally selected for qualitative analysis, one observational study [33] was excluded from the meta-analysis because it focused only on physical functioning as an HRQOL domain. We finally included 17 RCTs [34,35,36,37,38,39,40,41,42,43,44,45,46,47,48,49,50], five quasi-experiments [51,52,53,54,55], and three observational studies [56,57,58] in the meta-analysis.

### 3.2. Characteristics of Selected Studies

Table 1 summarizes the characteristics of the 26 selected studies, which were conducted in the USA [41], Europe [33,36,37,39,41,47,48,49,50,51,52,56], Australia [46,54], and Asia [34,35,38,40,42,44,45,53,55,57,58]. While one quasi-experimental study [55] and two observational studies [33,58] were conducted from 1998 to 2000, the 23 remaining studies [34,35,36,37,38,39,40,41,42,43,44,45,46,47,48,49,50,51,52,53,54,56,57] were conducted between 2010 and 2020.

A total of 4653 office workers from 17 RCTs (45.5%) [34,35,36,37,38,39,40,41,42,43,44,45,46,47,48,49,50], five quasi-experiments (13.0%) [51,52,53,54,55], and three observational studies (41.5%) [56,57,58] were pooled in the meta-analyses. Sample sizes of the included RCTs, quasi-experimental, and observational studies varied from 20 to 853, from 11 to 313, and from 109 to 1017 participants, respectively.

Of the 17 RCTs, three were pilot [34,37,40], three were cluster [36,43,45], and one was a cross-over trial [49]. Most RCTs (11 of 17) [34,35,37,38,39,40,41,44,45,47,50] recruited office workers with health problems; most quasi-experimental studies (three of five) [52,54,55] recruited healthy office workers; and most observational studies (three of four) [33,57,58] recruited both types of office workers. Of the 12 studies that recruited office workers with health problems, 10 studies [34,35,37,39,40,41,44,45,47,50] focused on musculoskeletal disorders, including neck, shoulder, and low back pain.

Various physical exercise interventions including walking, yoga, tai chi, aerobics, neck movements, vibration training, stretching, strengthening, flexibility exercises, endurance training, and non-purposeful movements were used in the RCTs and quasi-experimental studies. The duration of intervention ranged from 5 to 48 weeks, with each physical exercise session lasting from 3 to 150 min. The frequencies of indirectly supervised and unsupervised sessions were more flexible than those of directly supervised sessions, which varied from 10 to 60 min per session and mostly from two to three sessions per week. The most common type of intervention used was directly supervised physical exercise [35,37,38,41,42,43,44,46,47,51,52]. The dropout rates of participants in studies using directly supervised, indirectly supervised, and unsupervised physical exercise ranged from 5.26% to 36.76%, from 0% to 43.14%, and from 0% to 16.28%, respectively. There was a lack of description for physical exercise among observational studies.

While the effects of physical exercise on HRQOL in office workers were conflicting in the included RCTs and quasi-experimental studies, the four observational studies [33,56,57,58] found consistent and positive associations. Most RCTs recruiting healthy office workers reported no significant differences in HRQOL scores between the intervention and control groups [36,43,46,48]. A similar association was observed in RCTs with indirectly supervised interventions [36,40,43,45]. Compared with control groups, office workers who performed directly supervised [35,38,41,42,44,47,51] or unsupervised [35,49,50,53,55] physical exercise mostly experienced significant improvements in HRQOL.

### 3.3. ROB of Selected Studies

Appendix A, Table A4 shows the ROB assessments for the 26 included studies. The overall quality of 17 RCTs raised some potential concerns, mostly resulting from potential ROBs relating to deviations produced from intended interventions (for all 17) and measurement of the outcome (for 11 among 17). Although all RCTs [34,35,36,37,38,39,40,41,42,43,44,45,46,47,48,49,50] were rated as low ROB for randomization process and selection of the reported results, nine RCTs [36,37,38,41,42,44,46,47,49] raised some concerns about missing outcome data.

We assessed ROB as low for five quasi-experimental studies [51,52,53,54,55] for participant selection, classification of interventions, and selection of reported results. However, the study by Genin et al. [52] was judged to be at serious overall ROB because of missing data. Potential sources of baseline confounding bias and bias arising from the measurement of outcomes resulted in moderate overall ROBs in the four remaining studies [51,53,54,55].

All four observational studies [33,56,57,58] were of moderate quality. We identified ROB as low for three cross-sectional studies [56,57,58] in the ascertainment of exposure, comparability based on design and analysis, and statistic test domains, but the studies provided insufficient descriptions of the response rate or the characteristics of the responders and the non-responders. Since the cohort study by Stafford et al. [33] used a self-reported questionnaire, we identified potential sources of exposure ascertainment and outcome assessment ROB.

### 3.4. Effects of Physical Exercise on HRQOL in Office Workers

Three multi-arm RCTs by Shariat et al. [35], Taylor et al. [43], and Salo et al. [50] contributed a total of six intervention–control comparisons to the meta-analyses because each study involved two intervention groups and one control group. From the RCT by Caputo et al. [37], which compared neck–shoulder resistance training with stretching and postural exercises, we extracted pre- and post-intervention data of each group as a comparison pair and treated each comparison pair separately because our primary purpose was to compare physical exercise interventions with controls. The quasi-experimental study by Genin et al. [52] also provided data for two pre- and post-intervention comparisons. Thus, we obtained 21 and six comparisons from 17 RCTs and five quasi-experimental studies, respectively.

**Figure 2:**
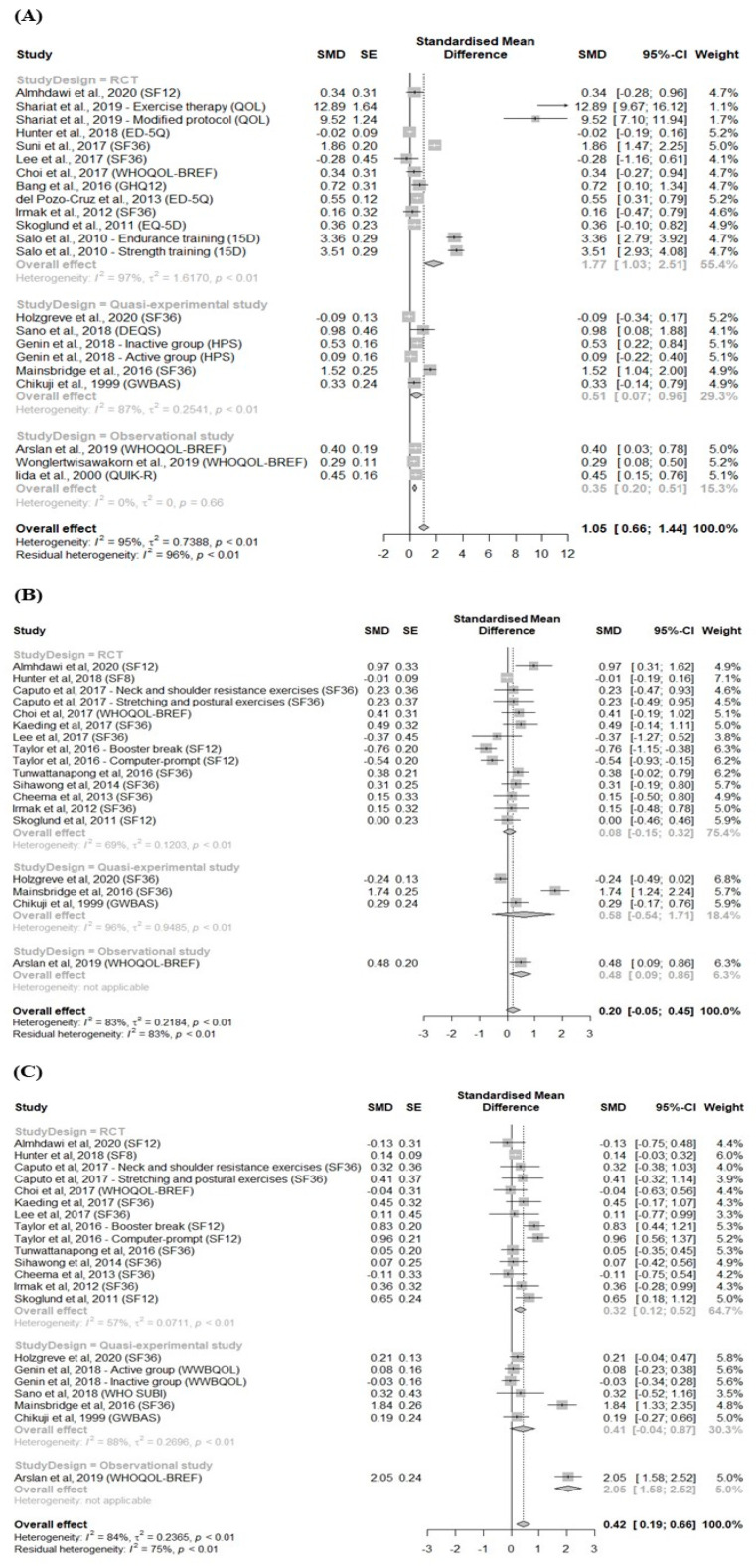
Meta-analyses of the effects of physical exercise on three HRQOL domains in office workers. (**A**) General; (**B**) physical; and (**C**) mental. HRQOL = health-related quality of life; SMD = standardized mean difference; SE = standard error; CI = confidence interval.

Forest plots in Figure 2 display results of the meta-analyses for the effects of physical exercise on each HRQOL domain. Compared with the control groups, the exercise groups showed statistically significant improvements in both general and mental HRQOL (SMD = 1.05; 95% CI: 0.66 to 1.44 for general and SMD = 0.42; 95% CI: 0.19 to 0.66 for mental HRQOL), although there was extreme heterogeneity among the studies (I^2^ = 95%, *p* < 0.01 for general and I^2^ = 84%, *p* < 0.01 for mental HRQOL). The pooled effect size for physical HRQOL was slightly positive but not statistically significant (SMD = 0.20, 95% CI: −0.05 to 0.45).

For general HRQOL, the pooled SMD obtained from the RCTs (SMD = 1.77; 95% CI: 1.03 to 2.51) was larger than those of the quasi-experimental and observational studies (SMD = 0.51; 95% CI: 0.07 to 0.96 for quasi-experimental studies and SMD = 0.35; 95% CI: 0.20 to 0.51 for observational studies). We observed opposite patterns in the physical and mental domains. For physical HRQOL, the pooled SMDs (95% CIs) obtained from RCT, quasi-experimental, and observational studies were 0.08 (−0.15 to 0.32), 0.58 (−0.54 to 1.71), and 0.48 (0.09 to 0.86), respectively, while for mental HRQOL, the SMDs were 0.32 (0.12 to 0.52), 0.41 (−0.04 to 0.87), and 2.05 (1.58 to 2.52), respectively.

### 3.5. Effects of Physical Exercise on HRQOL by Subgroups of Office Workers

Table 2 shows results of subgroup analyses by office worker characteristics and types of intervention only using data obtained from RCTs. After physical exercise interventions, compared with healthy office workers, unhealthy office workers experienced greater improvements in the general and physical domains (SMD = 2.76; 95% CI: 1.63 to 3.89 in unhealthy workers and SMD = 0.23; 95% CI: −0.09 to 0.56 in healthy workers for general HRQOL; SMD = 0.38; 95% CI: 0.17 to 0.58 in unhealthy workers and SMD = −0.20; 95% CI: −0.51 to 0.11 in healthy workers for physical HRQOL) but a smaller improvement in the mental domain (SMD = 0.12; 95% CI: −0.08 to 0.33 in unhealthy workers and SMD = 0.49; 95% CI: 0.13 to 0.84 in healthy workers). Unhealthy office workers were extremely heterogeneous in the general HRQOL analysis (I^2^ = 97%, *p* < 0.01) but homogeneous in the physical and mental HRQOL analyses (I^2^ = 0%, *p* = 0.49 and I^2^ = 0%, *p* = 0.88, respectively).

Both unsupervised and directly supervised physical exercise significantly improved general HRQOL (SMD = 3.35; 95% CI: 1.42 to 5.28 for unsupervised exercise and SMD = 1.77; 95% CI: 0.73 to 2.81 for directly supervised exercise), with extreme heterogeneity (I^2^ = 98% and *p* < 0.01 for unsupervised exercise and I^2^ = 95% and *p* < 0.01 for directly supervised exercise), but neither improved the physical domain (SMD = 0.17; 95% CI: −0.15 to 0.48 for unsupervised exercise and SMD = 0.08; 95% CI: −0.38 to 0.55 for directly supervised exercise). A significant improvement in the mental domain was observed in office workers who performed unsupervised physical exercise (SMD = 0.52; 95% CI: 0.20 to 0.84), with low heterogeneity (I^2^ = 0% and *p* = 0.74). There were no significant associations between indirectly supervised physical exercise and the three HRQOL domains.

### 3.6. Verification of Analysis Results

Seventeen RCT, five quasi-experimental, and three observational studies provided data for 21 comparisons, 6 comparisons, and 3 associations, respectively, all of which were used to calculate a total of 30 effect sizes. Leave-one-out analyses were performed by omitting each effect size in turn in the three primary meta-analyses (Table 3). Pooled SMDs for general, physical, and mental HRQOL ranged from 0.88 to 1.13, 0.10 to 0.27, and 0.34 to 0.45, respectively. All SMDs for the general and mental HRQOL domains were statistically significant, while those for the physical domain were not.

**Figure A1:**
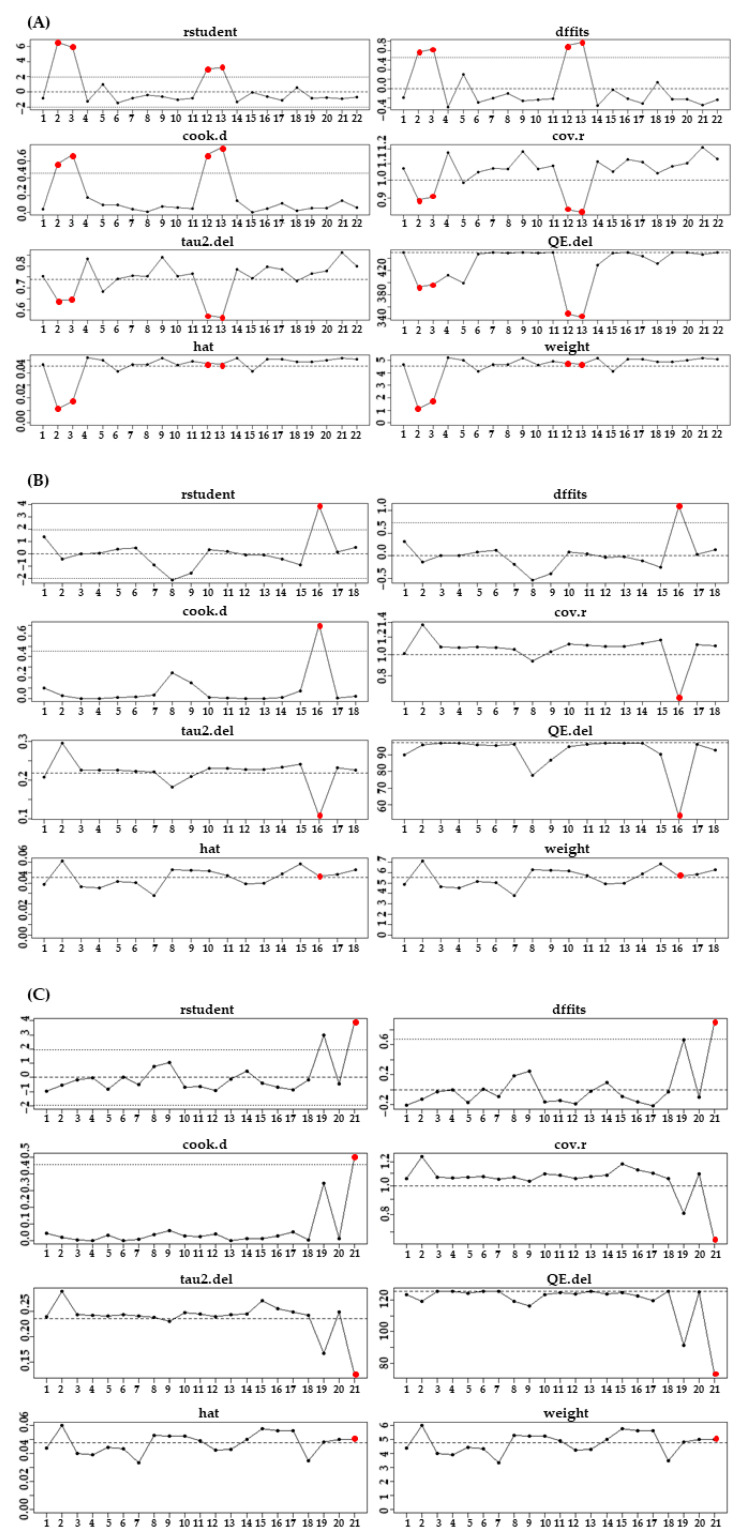
Influence sensitivity analyses result in meta-analyses for the effects of physical exercise on three HRQOL domains in office workers. (**A**) General; (**B**) physical; and (**C**) mental. Comparisons that exerted a very large influence on the overall effect sizes are highlighted in red. HRQOL = health-related quality of life.

After removing four [35,50], one [54], and one [56] outliers detected in influence sensitivity analyses for general, physical, and mental HRQOL (Appendix A, Figure A1), we re-performed the three primary meta-analyses. The pooled SMDs were then 0.47 (95% CI: 0.24 to 0.70) for general, 0.10 (95% CI: −0.10 to 0.29) for physical, and 0.34 (95% CI: 0.15 to 0.53) for mental HRQOL (Table 3).

### 3.7. Publication Bias

**Figure A2:**
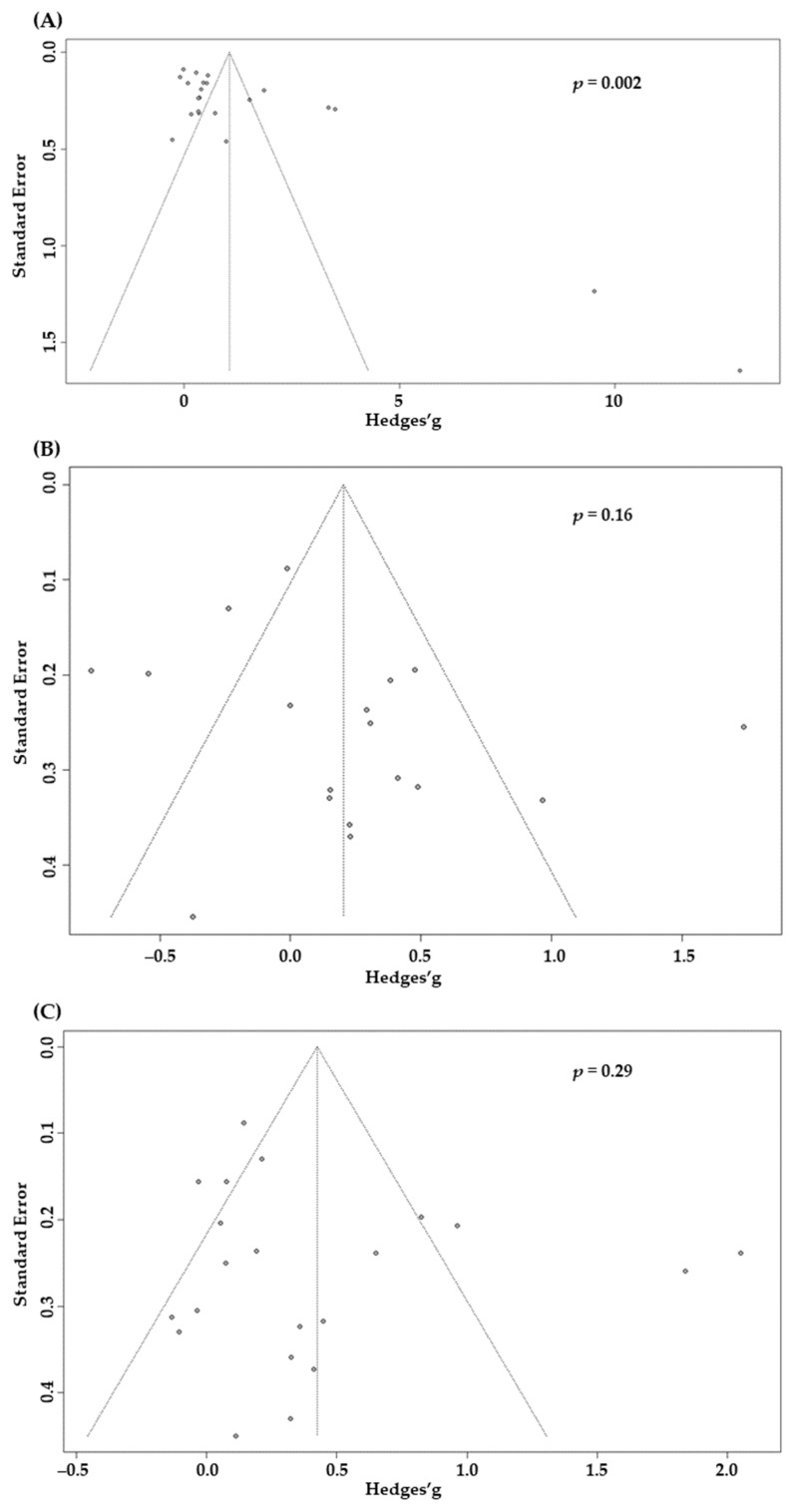
Funnel plots and *p*-values obtained from Egger’s test in meta-analyses for the effects of physical exercise on three HRQOL domains in office workers. (**A**) General; (**B**) physical; and (**C**) mental. HRQOL = health-related quality of life.

The meta-analyses for the effects of physical exercise on the physical and mental HRQOL domains showed visual evidence in symmetrical funnel plots and non-significance on Egger’s regression asymmetry tests (*p* = 0.16 for physical HRQOL and 0.29 for mental HRQOL). However, asymmetry was observed in the general domain (*p* = 0.002; Appendix A, Figure A2).

## 4. Discussion

A variety of physical exercise interventions have been developed to improve HRQOL in office workers, and we found significant and positive effects of physical exercise on general and mental HRQOL. The association between exercise and physical HRQOL was positive but small and not significant. After physical exercise interventions, unhealthy office workers experienced greater improvements in general and physical HRQOL but a smaller improvement in mental HRQOL than did healthy office workers. Unsupervised physical exercise significantly improved general and mental but not physical HRQOL. Significant improvement in general HRQOL was observed in office workers who performed directly supervised physical exercise, but there were no significant associations between indirectly supervised exercise and the three HRQOL domains.

To assess the overall effect of physical exercise on HRQOL in office workers, we pooled data obtained from not only RCTs and quasi-experiments but also observational studies in which the researchers adequately controlled for potential confounders in the design or analysis. Due to the extreme heterogeneity in the three primary analyses, we performed subgroup analyses by study design, and we observed considerable differences among sub-effect sizes obtained from the RCT, quasi-experimental, and observational studies. These differences could be due to the fact that the evidence levels differed among study designs. For this reason, we used data only from RCTs, regarded as the highest level of evidence, in the subgroup analyses by office worker characteristics and intervention types, to minimize the methodological heterogeneity among the included studies.

The three primary meta-analyses showed significant improvements in general and mental HRQOL but a non-significant improvement in physical HRQOL. Although the effects of physical exercise on general HRQOL in office workers were conflicting among the included studies, the overall effect size was large and significant. On one hand, the three negative SMDs obtained from the included studies [36,40,51] were very small and not significant. Of these, results obtained from the cluster RCT by Hunter et al. [36] and from the pilot study by Lee et al. [40] needed to be interpreted with caution because of low power. The high dropout rates of participants in the two studies could also affect the interpretation [36,51]. Furthermore, the three cross-sectional studies [56,57,58] found a consistent and significant positive association between physical exercise and general HRQOL despite small effect sizes ranging from 0.29 to 0.45. On the other hand, considering three RCTs that contributed the most to the large and significant overall effect size [35,41,50], all of them recruited office workers with musculoskeletal pains. This was in line with previous reviews by Gobbo et al. [14] and Louw et al. [15], showing that after doing physical exercise, office workers with neck or low back pain experienced a significant decrease in pain symptoms, resulting in better general HRQOL. Our results from subgroup analyses by office worker characteristics confirmed that the effect of physical exercise on general HRQOL was much larger in unhealthy office workers than in healthy workers.

Most SMDs showing the association between physical exercise and mental HRQOL obtained from the included studies were small and positive but not significant, whereas the overall effect size was significant. This was primarily contributed by large and significant SMDs obtained from the cluster RCT by Taylor et al. [43], the quasi-experimental study by Mainsbridge et al. [54], and the observational study by Arslan et al. [56]. It is worth noting that these studies all recruited office workers without any health problems and had low dropout rates despite using different types of physical exercise interventions. To understand further the differences in the effects of physical exercise on mental HRQOL between office workers with and without health problems, we performed a subgroup analysis and found that healthy office workers experienced a greater improvement compared with unhealthy office workers. Indeed, a systematic review by Abdin et al. [13] suggested that office workers could improve their mental HRQOL by participating in any form of physical exercise in an office setting. Physical exercise benefited mental health by reducing negative mood, depression, and anxiety, and by improving cognitive function and self-esteem [59]. From a physiological perspective, investigators have reported several changes in neurophysiology and level of neurochemical markers such as lactate, cortisol, neurotransmitters (dopamine, norepinephrine, serotonin, acetylcholine, gamma aminobutyric acid, and glutamate), and neurotrophins (brain-derived neurotrophic factor, insulin-like growth factor 1, and vascular endothelial growth factor) after acute bouts of exercise [60]. Our findings strengthened the known positive association between physical exercise and mental HRQOL in office workers and newly discovered that the association between the two was stronger in healthy office workers than in unhealthy workers. Along with the findings of Gill et al., suggesting that social and emotional benefits could be primary motivators for preventing adverse outcomes of workers [61], our results support that physical exercise should be widely encouraged among office workers.

The small and non-significant association between physical exercise and physical HRQOL may be attributable to the conflicting SMDs obtained from the included studies. While some SMDs were positive and significant [34,54,56], some were negative and significant [43], and the rest were small and not significant. The subgroup analysis by office worker characteristics showed that there was a significant improvement in physical HRQOL among unhealthy office workers and no improvement among healthy office workers after physical exercise interventions. This could be partially explained by the ways in which HRQOL surveys were administered. In our review, the physical domain was predominantly measured by summarizing four sub-domains of the 36-Item Short-Form Health Survey, three of which were physical functioning, role limitations due to physical health, and pain. For healthy office workers, their physical conditions before physical exercise intervention did not limit them in their daily activities. Therefore, they might experience few changes relating to the physical sub-domains after physical exercise interventions, even if the interventions improved their physical fitness. In contrast, unhealthy office workers, most of whom were experiencing musculoskeletal disorders or any related limitations, experienced greater improvements. Although it is widely accepted that physical exercise improves physical HRQOL in office workers, our findings suggest that further studies are needed to determine the optimal intensity and type of physical exercise for office workers with different health conditions.

Since the physical exercise interventions were very diverse such as stretching, strengthening, flexibility exercises, etc. with different exercise intensities, the number of studies included in the meta-analysis for the effect of each type of intervention on HRQOL was very small, leading to low power. For this reason, we classified the diverse interventions into three groups (i.e., unsupervised, directly supervised, and indirectly supervised) and performed subgroup analyses to investigate how different the effect of physical exercise on the three HRQOL domains was depending on the three groups. General HRQOL was improved by unsupervised or directly supervised interventions, while mental HRQOL was improved only by unsupervised physical exercise intervention. However, there were no significant associations between indirectly supervised physical exercise and any of the three domains. The dropout rates seemed to be the lowest in the studies with unsupervised physical exercise interventions, but all the included studies inadequately described at which levels participants adhered to the interventions. Consistent with our subgroup analysis, Gobbo et al. [14] found a marked reduction in pain symptoms after supervised exercise programs in office workers with low back pain; indeed, exercising under direct supervision by professional instructors ensured the correct postures and made strong exercise intensity. However, office workers might not prefer to exercise during their lunch break, or they might feel embarrassed to exercise in public or with colleagues; even strict supervision or regular reminders via phone notifications, application reminders, or text messages potentially caused stress and discomfort [14]. All of these could explain the lack of significant associations between directly or indirectly supervised physical exercise interventions and mental HRQOL. In our review, unsupervised interventions mostly took place in two steps—short training guided by professionals that lasted from 3 to 12 days, and then interventions conducted at home that were completely unguided or guided by instructional videos. Despite the positive effects of unsupervised physical exercise on general and mental HRQOL, the diversity in physical exercise types and durations led to difficulties in generating conclusive recommendations. Therefore, future research studies are needed to address this gap in knowledge.

This review has several limitations. First, the overall heterogeneity among studies was large in all three primary meta-analyses. Although subgroup analyses in our study were conducted using random-effects models, the heterogeneity remained in some analyses, limiting the interpretation of our findings. Second, we identified considerable ROBs for most of the included studies, primarily because it is nearly impossible to apply a blinded physical exercise intervention, which gives no information about the interventions to the participants or the instructors. Moreover, most questionnaires measuring HRQOL outcomes were self-reported. It could reduce the size of associations between physical exercise and HRQOL because of random errors due to wrong memory. Third, we used several statistical tools to estimate means and SDs and calculate Hedge’s g values because of a variety of indicators for effect sizes used in different studies. Although this was the only way to avoid missing data, it could have resulted in deviations. Fourth, because of a great variety of physical exercise interventions with different intensities used in the included studies, we could not clarify the effect of each specific type of intervention on the three HRQOL domains.

Despite these limitations, we conducted a comprehensive search without English-language restriction, reducing potential systemic bias. Pooling data obtained from multiple study designs provided an overview of the effects of physical exercise on three main domains, and leave-one-out and influence sensitivity analyses confirmed the robustness of our findings. Subgroup analyses identified factors that affected the relationships between physical exercise and the different HRQOL domains, providing new evidence for future research.

## 5. Conclusions

This systematic review summarized the currently available evidence on the association between physical exercise and HRQOL in office workers. The meta-analyses indicated that physical exercise significantly improved general and mental HRQOL while having a positive but small and non-significant effect on physical HRQOL in office workers. Compared with healthy office workers, unhealthy office workers experienced greater improvements in general and physical HRQOL but a smaller improvement in mental HRQOL. Unsupervised and directly supervised physical exercise significantly improved general HRQOL, but neither improved the physical HRQOL. There was a significant association between unsupervised physical exercise and mental HRQOL, but no significant associations between indirectly supervised physical exercise and any HRQOL domains in office workers. Therefore, physical exercise, especially unsupervised physical exercise, should be encouraged to improve HRQOL in office workers. However, detailed recommendations could not be made because the exercise type in the included studies was very diverse with different exercise intensities. Further studies are needed to determine the optimal exercise for office workers with different health conditions.

## Figures and Tables

**Table 1:** Characteristics of the 26 included studies.

17 Randomized Controlled Trials												
Study	Office Worker Characteristics ^1^	N	Country	Dropout Rate	Intervention				Control	HRQOL Scale		Main Findings Relating to HRQOL
					Description ^2^	Frequency ^3^	Duration ^3^	Ni	Description	Nc		
Almhdawi et al. [34]	Low back pain; 30–55 y	41	Jordan	4.88% (2/41)	Stretching and strengthening. IS	NI	6 w	21	Nutrition application	20	SF12	Compared with the CG, the IG demonstrated significant increase in physical component.
Shariat et al. [35]	Chronic lower back pain; 20–50 y	76	Iran	5.26% (4/76)	(1) Exercise therapy. US (2) Modified: relaxation training followed by the PE. DS	~45 min/s; 3 s/w	6 w	(1) 19 (2) 19	No intervention	19	QOL	Significant differences in HRQOL were found between the two IGs and CG.
Hunter et al. [36]	Healthy; 43.6 ± 9.6 y	853	Ireland	43.14% (368/853)	Walking. IS	150 min/w	24 w	457	No intervention	396	EQ-5D SF8	After adjusting for baseline values, there were no significant differences between groups for HRQOL.
Caputo et al. [37]	Chronic neck pain; 28–51 y	35	Spain	22.86% (8/35)	(1) Neck-shoulder resistance. DS (2) Stretching and postural exercises. DS	45 min/s; 2 s/w	7 w	18	Pre-intervention	17	SF36	SF36 scores remained unchanged.
Choi et al. [38]	Having ≥2 risk factors for MS; 40–60 y	68	South Korea	36.76% (25/68)	Tai chi plus health education on MS. DS	60 min/s; 2 s/w	12 w	34	Health education on MS	34	WHOQOL-BREF	There were significant improvements in the physical and environmental domains and no significant changes in the psychological and social domains.
Kaeding et al. [39]	Chronic low-back pain; 45.5 ± 9.1 y	41	Germany	4.89% (2/41)	Whole-body vibration training. US	~15 min/s; 2.5 s/w	12 w	21	Usual activity	20	SF36	Positive effects in the IG compared with the CG regarding the SF36 were not significant.
Lee et al. [40]	Chronic neck pain; 25–35 y	20	South Korea	5% (1/20)	6 neck movements. IS	~15min/s; 2s/w	8 w	11	Provided a brochure	9	SF36	There were no significant differences in SF-36 scores between IG and CG.
Suni et al. [41]	Neck or/and lower back pain; 30–50 y	170	Finland	11.18% (19/170)	Flexibility, strength and core exercises. DS	60 min/s; 2 s/w	10 w	87	Usual activity	83	SF36	HRQOL in terms of pain and physical functioning improved.
Bang et al. [42]	Healthy; 39.8 ± 10.4 y	60	South Korea	25% (15/60)	Walking. DS	40 min/s; 2 s/w	5w	30	No intervention	30	GHQ12	The urban forest walking program had positive effects on HRQOL.
Taylor et al. [43]	Healthy; ≥18 y	185	USA	5.4% (10/185)	(1) Booster Break. DS (2) Computer-Prompt: walking. IS	(1) ~15 min/s/d; (2) 3 min/s; 5 s/d	24 w	(1) 76 (2) 61	Usual breaks	48	SF12	No main effects were observed among HRQOL variables in any of the three study arms.
Tunwattanapong et al. [44]	Neck/shoulder pain; 36.5 ± 8.7 y	96	Thailand	9.38% (9/96)	Stretching. DS	10–15 min/s; 2 s/d; 5 d/w	4 w	48	Provided a brochure	48	SF36	The improvement was greater in the IG than in the CG for physical dimension of the SF36.
Sihawong et al. [45]	Low back pain; 18–55 y	76	Thailand	0% (0/76)	Stretching and endurance training. IS	4 s/w	48 w	23	No intervention	53	SF36	There was no significant difference in HRQOL between the IG and the CG.
Cheema et al. [46]	Healthy; 38 ± 12 y	37	Australia	8.1% (3/37)	Hatha yoga. DS	50 min/s; 3 s/w	10 w	18	Usual activity	19	SF36	None of the HRQOL domain scores changed significantly in the IG vs. CG
del Pozo-Cruz et al. [47]	Low back pain; 18–64 y	100	Spain	10% (10/100)	Strengthening, flexibility, mobility and stretching. DS	11 min/s/d	36 w	50	Usual care	50	EQ-5D	There were improvements in most of the EQ-5D components in the IG compared with the CG.
Irmak et al. [48]	Healthy; 20–65 y	39	Turkey	0% (0/39)	Strengthening and stretching. US	45 min/s	10 w	20	No intervention	19	SF36	There was no statistically significant difference between the IG and the CG.
Skoglund et al. [49]	Healthy; 42–54 y	42	Sweden	11.9% (5/42)	6-week qigong group training. US	17–25 min/s/d	13 w	37	Daily regular work	37	SF12 EQ-5D	The mental HRQOL domain was significantly improved in IG compared to CG.
Salo et al. [50]	Neck pain; 46 ± 6 y	180	Finland	1.67% (3/180)	(1) Strength training; (2) Endurance training. US	20 min/s; 3 s/w	48 w	(1) 60 (2) 60	Provided written information	60	15D	There was a significant improvement in the 15D total scores in both IGs. No changes occurred in the CG.
5 Quasi-experimental studies												
Study	Office worker characteristics ^1^	N	Country		Dropout rate	Description ^2^	Frequency ^3^	Duration	HRQOL scale	Main findings relating to HRQOL		
Holzgreve et al. [51]	Healthy or pain; 43.37 ± 11.24 y	(1) IG: 216 (2) CG: 97	Germany		19.17% (60/313)	Stretching. DS	10 min/s; 2 s/w	12w	SF36	Significantly improved outcomes in some HRQOL domains after the intervention.		
Genin et al. [52]	Healthy; 44.2 ± 9.8 y	(1) Active: 98; (2) Inactive: 95	France		25.39% (49/193)	Strengthening, stretching, cardiorespiratory. DS	2–3 s/w ≥45 min/s	20w	HPS WWBQOL	No significant difference was observed.		
Sano et al. [53]	Dry eye; 48.5 ± 11.0 y	11	Japan		0% (0/11)	Education and core strength training. US	3 s/w	10w	DEQS WHO SUBI	The DEQS scores significantly improved. The WHO SUBI scores did not change.		
Mainsbridge et al. [54]	Healthy; 43.81 ± 9.94 y	43	Australia		0% (0/43)	Non-purposeful movement. IS	Every 45 min	26w	SF36	The IG increased their HRQOL from pre-test to post-test with a medium effect size.		
Chikuji et al. [55]	Healthy; 47.8 ± 5.0 y	43	Japan		16.28% (7/43)	Short-term low intensity aerobic training. US	205 ± 117 min/2–3 s/w	8w	GWBAS	HRQOL improved significantly.		
4 Observational studies												
Study	Office worker characteristics ^1^	N	Country		Dropout rate	Study design	HRQOL scale	Main findings relating to HRQOL				
Arslan et al. [56]	Healthy; 25–60 y	109	Turkey		0%	Cross-sectional	WHOQOL-BREF	All subscales of the WHOQOL-BREF were significantly higher in office workers who did regular PE than in those who did not.				
Wonglertwisawakorn [57]	Both healthy and unhealthy; 40.20 ± 9.08 y	805	Thailand		38.51% (310/805)	Cross-sectional	WHOQOL-BREF	From multivariate analysis, the risk factors for poor quality of life were bachelor’s degree, single, absence of exercise, abnormal stress and high physical job demand. One protective factor for poor quality of life was high job control.				
Iida et al. [58]	Both healthy and unhealthy; 52 ± 9 y	1017	Japan		0%	Cross-sectional	QUIK-R	There were significant correlations between the total HRQOL and the subject’s age, sex, smoking habit, sleeping time, and PE.				
Stafford et al. [33]	Both healthy and unhealthy; 35–55 y	10,308	The UK		19% (1959/10,308)	Cohort	SF-36	Multiple logistic regression showed that PE, body mass index, fibrinogen, and insulin were independently associated with the physical functioning domain.				

N = total number of participants; Ni = number of participants in the intervention group; Nc = number of participants in the control group; HRQOL = health-related quality of life; PE = physical exercise; NI = no information; IG = intervention group; CG = control group; MS = metabolic syndrome; SF12 = 12-Item Short-Form Health Survey; QOL = Quality of Life Questionnaire; EQ-5D = EuroQol Five-Dimensional Questionnaire; SF8 = 8-Item Short-Form Health Survey; SF36 = 36-Item Short-Form Health Survey; WHOQOL-BREF = World Health Organization Quality of Life Questionnaire–Brief Version; GHQ12 = 12-Item General Health Questionnaire; 15D = 15-Dimensional Health-related Quality of Life Measure; HPS = Health Perception Scale; WWBQOL = Worksite Well-Being and Quality of Life Questionnaire; DEQS = Dry Eye-related Quality of Life Score; WHO SUBI = World Health Organization’s Subjective Well-Being Inventory; GWBAS = General Well-Being Adjustment Scale; QUIK-R = Self-Completed Questionnaire for Quality of Life Revised; ^1^ y = years old; ^2^ IS = indirectly supervised; US = unsupervised; DS = directly supervised; ^3^ d = day; min = minute; s = session; w = week.

**Table 2:** The effects of physical exercise on three HRQOL domains in office workers by office worker characteristics and intervention types.

	k	Pooled SMD (95% CI)	Percentage (%)	Heterogeneity	
				I^2^ (%)	*p*-Value
General HRQOL domain		1.77 (1.03 to 2.51)	100	97	<0.01
Office worker characteristics					
Healthy	4	0.23 (−0.09 to 0.56)	33.8	56	0.08
Unhealthy	9	2.76 (1.63 to 3.89)	66.2	97	<0.01
Types of intervention					
Directly supervised	5	1.77 (0.73 to 2.81)	38.4	95	<0.01
Indirectly supervised	3	−0.002 (−0.17 to 0.16)	24.8	0	0.45
Unsupervised	5	3.35 (1.42 to 5.28)	36.8	98	<0.01
Physical HRQOL domain		0.08 (−0.15 to 0.32)	100	69	<0.01
Office worker characteristics					
Healthy	6	−0.20 (−0.51 to 0.11)	49.2	73	<0.01
Unhealthy	8	0.38 (0.17 to 0.58)	50.8	0	0.49
Types of intervention					
Directly supervised	6	0.08 (−0.38 to 0.55)	41.4	77	<0.01
Indirectly supervised	5	0.05 (−0.37 to 0.47)	37.9	78	<0.01
Unsupervised	3	0.17 (−0.15 to 0.48)	20.7	0	0.46
Mental HRQOL domain		0.32 (0.12 to 0.52)	100	57	<0.01
Office worker characteristics					
Healthy	6	0.49 (0.13 to 0.84)	51.0	79	<0.01
Unhealthy	8	0.12 (−0.08 to 0.33)	49.0	0	0.88
Types of intervention					
Directly supervised	6	0.27 (−0.07 to 0.61)	40.5	56	0.04
Indirectly supervised	5	0.26 (−0.12 to 0.65)	39.6	74	<0.01
Unsupervised	3	0.52 (0.20 to 0.84)	19.9	0	0.74

HRQOL = health-related quality of life; k = number of comparisons; SMD = standardized mean difference; CI = confidence interval.

**Table 3:** Sensitivity analyses for the effects of physical exercise on three HRQOL domains in office workers.

Study Omitted	General HRQOL Domain		Physical HRQOL Domain		Mental HRQOL Domain	
	k	Pooled SMD (95% CI)	k	Pooled SMD (95% CI)	k	Pooled SMD (95% CI)
Leave-one-out analysis						
Randomized controlled trial						
Almhdawi et al. [34]	21	1.09 (0.69 to 1.49)	17	0.16 (−0.09 to 0.41)	20	0.45 (0.21 to 0.69)
Shariat et al. [35]—Exercise therapy	21	0.90 (0.54 to 1.27)	–	–	–	–
Shariat et al. [35]—Modified protocol	21	0.89 (0.52 to 1.26)	–	–	–	–
Hunter et al. [36]	21	1.13 (0.71 to 1.55)	17	0.22 (−0.07 to 0.51)	20	0.44 (0.18 to 0.71)
Caputo et al. [37]—Neck and shoulder resistance exercises	–	–	17	0.20 (−0.06 to 0.46)	20	0.43 (0.18 to 0.67)
Caputo et al. [37]—Stretching and postural exercises	–	–	17	0.20 (−0.06 to 0.46)	20	0.42 (0.18 to 0.67)
Choi et al. [38]	21	1.09 (0.69 to 1.49)	17	0.19 (−0.07 to 0.45)	20	0.45 (0.20 to 0.69)
Kaeding et al. [39]	–	–	17	0.19 (−0.07 to 0.45)	20	0.42 (0.18 to 0.67)
Lee et al. [40]	21	1.11 (0.71 to 1.51)	17	0.23 (−0.03 to 0.48)	20	0.44 (0.19 to 0.68)
Suni et al. [41]	21	0.99 (0.61 to 1.38)	–	–	–	–
Bang et al. [42]	21	1.07 (0.67 to 1.47)	–	–	–	–
Taylor et al. [43]—Booster break	–	–	17	0.27 (0.03 to 0.51)	20	0.40 (0.16 to 0.65)
Taylor et al. [43]—Computer-prompt	–	–	17	0.25 (0.0005 to 0.50)	20	0.39 (0.15 to 0.64)
Tunwattanapong et al. [44]	–	–	17	0.19 (−0.07 to 0.45)	20	0.44 (0.20 to 0.69)
Sihawong et al. [45]	–	–	17	0.20 (−0.06 to 0.46)	20	0.44 (0.20 to 0.69)
Cheema et al. [46]	–	–	17	0.21 (−0.05 to 0.47)	20	0.45 (0.20 to 0.69)
del Pozo-Cruz et al. [47]	21	1.10 (0.68 to 1.53)	–	–	–	–
Irmak et al. [48]	21	1.10 (0.70 to 1.50)	17	0.21 (−0.05 to 0.47)	20	0.43 (0.18 to 0.67)
Skoglund et al. [49]	21	1.09 (0.69 to 1.50)	17	0.22 (−0.05 to 0.48)	20	0.41 (0.17 to 0.66)
Salo et al. [50]—Endurance training	21	0.89 (0.53 to 1.24)	–	–	–	–
Salo et al. [50]—Strength training	21	0.88 (0.53 to 1.23)	–	–	–	–
Quasi-experimental study						
Holzgreve et al. [51]	21	1.13 (0.72 to 1.53)	17	0.24 (−0.03 to 0.50)	20	0.44 (0.18 to 0.69)
Genin et al. [52]—Active group	21	1.11 (0.71 to 1.52)	–	–	20	0.44 (0.19 to 0.70)
Genin et al. [52]—Inactive group	21	1.09 (0.68 to 1.51)	–	–	20	0.45 (0.20 to 0.70)
Sano et al. [53]	21	1.06 (0.66 to 1.45)	–	–	20	0.43 (0.18 to 0.67)
Mainsbridge et al. [54]	21	1.02 (0.63 to 1.42)	17	0.10 (−0.10 to 0.29)	20	0.35 (0.14 to 0.57)
Chikuji et al. [55]	21	1.10 (0.69 to 1.50)	17	0.20 (−0.06 to 0.46)	20	0.44 (0.19 to 0.68)
Observational study						
Arslan et al. [56]	21	1.10 (0.69 to 1.50)	17	0.19 (−0.08 to 0.45)	20	0.34 (0.15 to 0.53)
Wonglertwisawakorn [55]	21	1.12 (0.70 to 1.55)	–	–	–	–
Iiada et al. [58]	21	1.10 (0.69 to 1.51)	–	–	–	–
Influence analysis						
Shariat et al. [35], Salo et al. [50]	18	0.47 (0.24 to 0.70)	–	–	–	–
Mainsbridge et al. [54]	–	–	17	0.10 (−0.10 to 0.29)	–	–
Arslan et al. [56]	–	–	–	–	20	0.34 (0.15 to 0.53)

HRQOL = health-related quality of life; k = number of comparisons; SMD = standardized mean difference; CI = confidence interval; – = unavailable data.

## Appendix A

**Table A1:** Search terms used for literature searches.

Intervention	Outcome	Population
Exercise	Quality of life	Office worker
Physical activity	Life quality	White-collar worker
Yoga	Quality of well-being	Office staff
Tai chi	QOL	Office employee
Qi gong	HRQOL	Desk-based worker
Aerobics	HRQL	
Sport	QWB	
Walking		
Swimming		
Running		
Training		
Dancing		
Climbing		
Pilates		
Corresponding controlled vocabulary indexing terms were used.		

**Table A2:** Classification of quantitative HRQOL data.

Study	General HRQOL Domain	Physical HRQOL Domain	Mental HRQOL Domain
18 Randomized controlled trials			
Almhdawi et al. [34]	Total score of SF12	PCS of SF12	MCS of SF12
Shariat et al. [35]	Quality of life questionnaire	–	–
Hunter et al. [36]	Health state score of EQ-5D	PCS of SF8	MCS of SF8
Caputo et al. [37]	–	PCS of SF36	MCS of SF36
Choi et al. [38]	Total score of WHOQOL-BREF	Physical health score of WHOQOL-BREF	Psychological health score of WHOQOL-BREF
Kaeding et al. [39]	–	PCS of SF36	MCS of SF36
Lee et al. [40]	GH perception score of SF36	PCS of SF36	MCS of SF36
Suni et al. [41]	GH perception score of SF36	–	–
Bang et al. [42]	Total score of GHQ12	–	–
Taylor et al. [43]	–	PCS of SF12	MCS of SF12
Tunwattanapong et al. [44]	–	PCS of SF36	MCS of SF36
Sihawong et al. [45]	–	PCS of SF36	MCS of SF36
Cheema et al. [46]	–	PCS of SF36	MCS of SF36
del Pozo-Cruz et al. [47]	Visual analogical score of EQ-5D	–	–
Irmak et al. [48]	GH perception score of SF36	PCS of SF36	MCS of SF36
Skoglund et al. [49]	Visual analogical score of EQ-5D	PCS of SF12	MCS of SF12
Salo et al. [50]	Total score of 15D	–	–
4 Quasi-experimental studies			
Holzgreve et al. [51]	GH perception score of SF36	PCS of SF36	MCS of SF36
Genin et al. [52]	Total score of HPS	–	Total score of WWBQOL
Sano et al. [53]	Total score of DEQS	–	Positive well-being score of WHO SUBI
Mainsbridge et al. [54]	Total score of SF36	PCS of SF36	Mental component score of SF36
Chikuji et al. [55]	Total score of GWBAS	Physical symptoms score of GWBAS	Emotional status score of GWBAS
3 Observational studies			
Arslan et al. [56]	Total score of WHOQOL-BREF	Physical health score of WHOQOL-BREF	Psychological health score of WHOQOL-BREF
Wonglertwisawakorn [55]	Total score of WHOQOL-BREF	–	–
Iida et al. [58]	Total score of QUIK-R	–	–

HRQOL = health-related quality of life; SF12 = 12-Item Short-Form Health Survey; PCS = physical component score; MCS = mental component score; GH = general health; SF36 = 36-Item Short-Form Health Survey; EQ-5D = EuroQol Five-Dimensional Questionnaire; SF8 = 8-Item Short-Form Health Survey; WHOQOL-BREF = World Health Organization Quality of Life Questionnaire–Brief Version; GHQ12 = 12-Item General Health Questionnaire; 15D = 15-Dimensional Health-related Quality of Life Measure; HPS = Health Perception Scale; WWBQOL = Worksite Well-Being and Quality of Life Questionnaire; DEQS = Dry Eye-related Quality of Life Score; WHO SUBI = World Health Organization’s Subjective Well-Being Inventory; GWBAS = General Well-Being Adjustment Scale; QUIK-R = Self-Completed Questionnaire for Quality of Life Revised; – = unavailable data.

**Table A3:** Calculation of means and SDs using data extracted from the included studies and conversion of effect sizes to Hedges’s g.

Estimating Means and Standard Deviations (SDs) from Medians and Interquartile Ranges ^1^									
Study			Indicator	General HRQOL		Physical HRQOL		Mental HRQOL	
				IG	CG	IG	CG	IG	CG
Caputo et al. [37]									
Neck and shoulder resistance exercises			Sample size	–	–	14	18	14	18
			Median	–	–	51.2	50.4	49.3	41.8
			Quartile 1	–	–	45.0	40.3	36.3	29.8
			Quartile 3	–	–	55.6	54.3	53.2	50.9
			Mean	–	–	50.54	48.16	45.99	40.75
			SD	–	–	8.73	11.26	13.92	16.97
Stretching and postural exercises			Sample size	–	–	13	17	13	17
			Median	–	–	49.4	48.1	52.7	51.0
			Quartile 1	–	–	45.0	39.2	48.6	34.9
			Quartile 3	–	–	53.9	54.0	56.6	55.8
			Mean	–	–	49.44	47.02	52.63	46.92
			SD	–	–	7.39	11.96	6.64	16.90
Suni et al. [41] ^2^			Sample size	83	66	–	–	–	–
			Median	74.74	71.32	–	–	–	–
			Quartile 1	73.54	70.03	–	–	–	–
			Quartile 3	75.94	72.52	–	–	–	–
			Mean	74.74	71.29	–	–	–	–
			SD	1.81	1.89	–	–	–	–
Skoglund et al. [49]			Sample size	37	37	37	37	37	37
			Median	80	73	17	17	24	20
			Quartile 1	65	60	14	15	21	18
			Quartile 3	90	82	19	18	25	24
			Mean	78.22	71.58	16.64	16.64	23.29	20.71
			SD	19.28	16.97	3.86	2.31	3.08	4.63
Converting OR or Cohen’s d to Hedges’s g ^3^									
Study	OR	LogOR	$V_{LogOR}$	d	$V_{d}$	J	g	$V_{g}$	$SE_{g}$
Wonglertwisawakorn [55]	1.7	0.5	0.037	0.29	0.011	1.0	0.29	0.011	0.105
del Pozo-Cruz et al. [47]	2.73	1.0	0.05	0.55	0.015	1.0	0.55	0.015	0.122
Iida et al. [58]	2.3	0.8	0.08	0.45	0.024	1.0	0.45	0.024	0.156

HRQOL = health-related quality of life; SD = standard deviation; IG = intervention group; CG = control group; – = unavailable data; ^1^ All estimations were conducted using the method of Wan et al. for both normal and skewed data [29]; ^2^ These data were extracted from box plots using the Web Plot Digitizer [28]; ^3^ All conversions were conducted using the formula of Borenstein et al. [30].

**Table A4:** ROB assessment of the included studies.

17 Randomized Controlled Trials—Revised Cochrane Risk-Of-Bias Assessment Tool for Randomized Controlled Trial (ROB 2.0) ^1^									
Study	Randomization Process	Deviations from Intended Interventions		Missing Outcome Data	Measurement of the Outcome			Selection of the Reported Result	Overall
Almhdawi et al. [34]	Low	Some concerns		Low	Low			Low	Some concerns
Shariat et al. [35]	Low	Some concerns		Low	Some concerns			Low	Some concerns
Hunter et al. [36]	Low	Some concerns		Some concerns	Low			Low	Some concerns
Caputo et al. [37]	Low	Some concerns		Some concerns	Low			Low	Some concerns
Choi et al. [38]	Low	Some concerns		Some concerns	Low			Low	Some concerns
Kaeding et al. [39]	Low	Some concerns		Low	Some concerns			Low	Some concerns
Lee et al. [40]	Low	Some concerns		Low	Some concerns			Low	Some concerns
Suni et al. [41]	Low	Some concerns		Some concerns	Some concerns			Low	Some concerns
Bang et al. [42]	Low	Some concerns		Some concerns	Some concerns			Low	Some concerns
Taylor et al. [43]	Low	Some concerns		Low	Some concerns			Low	Some concerns
Tunwattanapong et al. [44]	Low	Some concerns		Some concerns	Low			Low	Some concerns
Sihawong et al. [45]	Low	Some concerns		Low	Some concerns			Low	Some concerns
Cheema et al. [46]	Low	Some concerns		Some concerns	Some concerns			Low	Some concerns
del Pozo-Cruz et al. [47]	Low	Some concerns		Some concerns	Low			Low	Some concerns
Irmak et al. [48]	Low	Some concerns		Low	Some concerns			Low	Some concerns
Skoglund et al. [49]	Low	Some concerns		Some concerns	Some concerns			Low	Some concerns
Salo et al. [50]	Low	Some concerns		Low	Some concerns			Low	Some concerns
5 Quasi-experimental studies—Risk-of-bias in non-randomized studies—of interventions tool (ROBINS-I) ^2^									
Study	Baseline confounding	Selection of participants	Classification of interventions	Deviation from intended interventions	Missing data	Measurement of outcomes		Selection of reported results	Overall
Holzgreve et al. [51]	Moderate	Low	Low	Moderate	Moderate	Moderate		Low	Moderate
Genin et al. [52]	Moderate	Low	Low	Low	Serious	Moderate		Low	Serious
Sano et al. [53]	Moderate	Low	Low	Low	Low	Moderate		Low	Moderate
Mainsbridge et al. [54]	Moderate	Low	Low	Low	Low	Moderate		Low	Moderate
Chikuji et al. [55]	Moderate	Low	Low	Low	Moderate	Moderate		Low	Moderate
4 Observational studies—Newcastle-Ottawa quality assessment scale (NOS)									
3 Cross-sectional studies—Adapted NOS for cross-sectional studies ^3^									
Study	Selection				Comparability	Outcome			Overall
	Representativeness of the sample *	Sample size *	Non-respondents *	Ascertainment of exposure **	Based on design and analysis **	Assessment of outcome **		Statistical test *	
Arslan et al. [56]	–	*	–	**	**	–		*	6/10
Wonglertwisawakorn [55]	–	–	–	**	**	*		*	6/10
Iida et al. [58]	*	*	–	*	**	*		*	7/10
1 Cohort study—NOS for cohort Studies ^4^									
Study	Selection				Comparability	Outcome			Overall
	Representativeness of the exposed cohort *	Non-exposed cohort *	Ascertainment of exposure *	Outcome of interest *	Based on design and analysis **	Assessment of outcome *	Long enough follow-up *	Adequacy of follow-up cohort *	
Stafford et al. [33]	*	*	–	*	**	–	*	*	7/9

^1^ Each domain was rated as low, some concerns, or high ROB. If all ROB domains of a study were rated as low, the study was judged to be at low ROB. If at least one domain reported some concerns, the study was judged to raise some concerns. If at least one domain was rated as high ROB or multiple domains reported some concerns in a way that substantially lowered confidence in the result, the study was judged as high ROB; ^2^ Each domain was rated as low, moderate, serious, critical ROBs or no information. If all ROB domains of a study were rated as low, the study was judged to be at low ROB. If at least one domain was rated as moderate, serious, or critical ROB, the study was judged as moderate, serious, or critical ROB, respectively; ^3,4^ Maximum numbers of stars that could be assigned to each ROB sub-domain were put next to their names; therefore, a study could receive a maximum of two and three stars for the comparability of the group and the outcomes of interest domains, respectively. For the study group selection ROB domain, a cross-sectional study and a cohort study could receive given a maximum of five and four stars, respectively. Overall study ROB was calculated by summing the numbers of stars awarded for each of the domains; higher scores denoted lower ROBs; – = no star assigned. ROB = risk-of-bias.
